# Light and water treatment during the early grain filling stage regulates yield and aroma formation in aromatic rice

**DOI:** 10.1038/s41598-020-71944-5

**Published:** 2020-09-09

**Authors:** Yuzhan Li, Luxin Liang, Xiaomeng Fu, Zifeng Gao, Hecheng Liu, Jiangtao Tan, Mouloumdema Pouwedeou Potcho, Shenggang Pan, Hua Tian, Meiyang Duan, Xiangru Tang, Zhaowen Mo

**Affiliations:** 1 State Key Laboratory for Conservation and Utilization of Subtropical Agro-Bioresources, College of Agriculture, South China Agricultural University, Guangzhou, 510642 China; 2 Scientific Observing and Experimental Station of Crop Cultivation in South China, Ministry of Agriculture and Rural Affairs, Guangzhou, 510642 China

**Keywords:** Plant physiology, Plant sciences, Plant stress responses, Abiotic, Drought, Light stress

## Abstract

The effect of light and water on aromatic rice remain largely unclear. A pot experiment was conducted to investigate the influences of light-water treatments (CK: natural light and well-watered conditions, WS: natural light and water-stressed conditions, LL: low light and well-watered conditions, LL-WS: low light and water-stressed treatment) on yield and 2-acetyl-1-pyrroline (2AP) formation in aromatic rice. Compared with CK, the light-water treatments decreased grain yield (10.32–39.19%) due to reductions in the filled grain percentage and total dry weight, in the regulation of biomass distribution, and in the attributes of gas exchange and antioxidant response parameters. The 2AP content in grains increased in the LL treatment (5.08–16.32%) but decreased in the WS treatment compared with that in CK. The changes in 2AP were associated with changes in 2AP formation-related traits and element content. Low light and water stress led to yield declines in aromatic rice, but low light alleviated the decrease in 2AP content caused by water stress.

## Introduction

Rice is one of the most important food crops worldwide. Aromatic rice has a higher grain quality than non-aromatic rice, and consumers prefer aromatic rice due to its pleasant smell^1,2^. Aromatic rice plays a significant role in international rice markets^3^. The global demand for aromatic rice is increasing^4^.

Many volatile compounds have been detected in aromatic rice^5–7^, of these, 2-acetyl-1-proline (2AP) is a determinant of the aromatic properties of aromatic rice^8,9^. Previous studies have suggested that proline is an important precursor for 2AP formation^10,11^. In addition, the ornithine, glutamate, γ-aminobutyric acid (GABA), ^Δ^1-pyrroline-5-carboxylate(P5C), ^Δ^1pyrroline-5-carboxylate synthetase (P5CS), ornithine aminotransferase (OAT) and proline dehydrogenase (PDH) are highly related to the biosynthesis of 2AP^12–16^. Moreover, some studies have reported that micronutrients such as Mn and Zn contribute to the synthesis of 2AP in aromatic rice^14,17^.

In addition to the effects of genotype, environmental factors and cultivation practices affect the accumulation of 2AP in aromatic rice^6^. A previous study reported that the 2AP content was negatively correlated with sunshine hours^18^. Shading promoted the accumulation of 2AP in aromatic rice^19,20^. However, shading can lead to the inhibition of the transportation of photosynthetic products which ultimately causes yield decline^21^. In addition, shading resulted in changes in the antioxidant defence of rice plants^22^. In previous studies, comparative transcriptome profiling was performed, and certain genes in rice that are expressed under low light were identified^23^.

Irrigation is important for crop production. Water stress reduces the photosynthesis rate, growth, and biomass production and thereby decreases grain yield^24–26^. In addition, water stress leads to increases in the production of reactive oxygen species (ROS) and changes in antioxidant parameters^27,28^, and the expression of a series of genes in response to drought stress has been assessed^29,30^. However, the accumulation of 2AP in aromatic rice is affected by irrigation practices^31^. The 2AP content in aromatic rice can be increased with alternate wetting and drying conditions^32^. Drought stress during the grain filling stage can enhance the accumulation of 2AP in aromatic rice^11^.

A previous study reported that the synthesis of 2AP was highly related to abiotic stresses^33^. Low light or water stress could lead to improved 2AP accumulation. However, the effects of light-water on aromatic rice remain largely unknown. In this study, two elite Chinese aromatic rice varieties, Xiangyaxiangzhan and Yuxiangyouzhan, were grown under four light-water treatments to explore how light and water regulate yield and 2AP formation in aromatic rice.

## Results

### Effects of the light-water treatments on yield and yield-related traits

Compared with CK, LL and LL-WS significantly decreased the grain yield in Xiangyaxiangzhan by 39.19% and 34.64%, respectively. WS, LL, and LL-WS significantly decreased the grain yield in Yuxiangyouzhan by 25.44%, 30.79%, and 29.42%, respectively, when compared to those under CK. The light-water treatments (WS, LL, and LL-WS) decreased the filled grain percentage, and a significant decrease compared to CK was detected under LL and LL-WS. The light-water treatments had no notable effect on the effective panicles or the 1,000-grain weight in either variety (Table 1).

**Table 1:** Effect of light-water treatment on rice yield and yield-related traits.

Treatment	Effective panicles per pot	Filled grain percentage (%)	1,000-grain weight (g)	Yield (g pot^−1^)
**Xiangyaxiangzhan**				
CK	24.00a	59.32a	16.38a	19.57a
WS	23.50a	53.74ab	15.98a	17.55a
LL	23.33a	46.28c	16.14a	11.90b
LL-WS	23.33a	48.06bc	15.62a	12.79b
Mean	23.54	51.85	16.03	15.45
**Yuxiangyouzhan**				
CK	20.33a	45.92a	18.82a	21.11a
WS	18.25a	40.26ab	18.44a	15.74b
LL	19.00a	38.39b	17.87a	14.61b
LL-WS	19.50a	29.28c	18.72a	14.90b
Mean	19.27	38.46	18.46	16.59

Within a column for each cultivar, means followed by different letters are significantly different according to LSD (0.05).

CK, natural light and well-watered treatment; WS, natural light and water-stressed treatment; LL, low light and well-watered treatment; LL-WS, low light and water-stressed treatment.

### Effect of the light-water treatments on organ dry weight

Compared with CK, WS, LL, and LL-WS resulted in reductions in total dry weight due to reductions in the dry weight of the stem sheath, panicle, and leaf, except for the dry weight of the Xiangyaxiangzhan leaves (Table 2).

**Table 2:** Effect of light-water treatment on plant dry weight (g pot^−1^).

Treatment	Stem sheath dry weigh		Leaf dry weight		Panicle dry weight		Total dry weight	
	AS	MS	AS	MS	AS	MS	AS	MS
**Xiangyaxiangzhan**								
CK	45.62a	42.40a	8.39a	5.38b	21.34a	26.74a	75.35a	74.52a
WS	38.21ab	38.66a	8.04a	5.84ab	19.29a	25.35a	65.53ab	69.85ab
LL	31.94b	37.28ab	9.18a	6.51a	15.28b	17.79b	56.40b	61.59bc
LL-WS	34.52b	30.86b	8.68a	6.26ab	15.07b	19.27b	58.27b	56.38c
Mean	37.57	37.30	8.57	6.00	17.74	22.29	63.89	65.59
**Yuxiangyouzhan**								
CK	55.33a	57.00a	11.25a	6.14a	19.86a	33.58a	86.44a	96.73a
WS	49.89ab	49.83ab	6.40c	5.03b	15.91b	23.33b	72.19b	78.19b
LL	43.41b	45.11b	9.30b	5.18ab	15.81b	26.05b	68.52b	76.34b
LL-WS	54.41a	46.02b	6.34c	2.81c	17.41b	22.65b	78.16ab	71.47b
Mean	50.76	49.49	8.32	4.79	17.25	26.40	76.33	80.68

Within a column for each cultivar, means followed by different letters are significantly different according to LSD (0.05).

CK, natural light and well-watered treatment; WS, natural light and water-stressed treatment; LL, low light and well-watered treatment; LL-WS, low light and water-stressed treatment.

### Effects of the light-water treatments on gas exchange parameters and SPAD value

Compared with CK, WS and LL-WS significantly decreased Pn in Xiangyaxiangzhan at AS (after shading). The LL and LL-WS significantly decreased Pn in Yuxiangyouzhan at AS. There was no significant difference in Pn among the treatments in either variety at MS (maturity stage). For Xiangyaxiangzhan, the Tr decreased substantially in response to light-water treatment at AS. For Yuxiangyouzhan, the WS and LL-WS caused significant reductions in the Tr at MS and AS, respectively. LL significantly improved the Tr in Yuxiangyouzhan at MS, and the Gs in Xiangyaxiangzhan was significantly decreased under the light-water treatments at AS. WS and LL-WS resulted in a marked reduction in Gs in Yuxiangyouzhan at AS and MS, while LL significantly increased Gs. For Xiangyaxiangzhan, the Ci showed a significant reduction under WS and LL-WS compared with that under CK at AS and MS, respectively. For Yuxiangyouzhan, LL and LL-WS significantly increased the Ci at AS compared with that under CK. WS and LL-WS substantially reduced the Ci, but LL significantly increased the Ci in Yuxiangyouzhan at MS. For Xiangyaxiangzhan, significant increase in the SPAD values at AS and MS compared with that under CK were observed in response to the light-water treatments. For Yuxiangyouzhan, WS significantly reduced the SPAD value at AS, while LL and LL-WS significantly increased the SPAD value at MS (Table 3).

**Table 3:** Effect of light-water treatment on gas exchange parameters and SPAD value.

Treatment	Pn (μ mol CO_2_ m^−2^ s^−1^)		Tr (mmol H_2_O m^−2^ s^−1^)		Gs (mol H_2_O m^−2^ s^−1^)		Ci (μmol CO_2_ mol^−1^)		SPAD value	
	AS	MS	AS	MS	AS	MS	AS	MS	AS	MS
**Xiangyaxiangzhan**										
CK	15.03a	7.93a	10.91a	4.79a	0.57a	0.15a	316.74a	276.67a	23.33c	15.15c
WS	13.01b	8.13a	7.73c	4.83a	0.34c	0.16a	297.76b	281.00a	27.73b	17.38b
LL	15.22a	7.69a	9.63b	5.21a	0.51b	0.15a	307.03ab	265.56a	31.05a	18.18b
LL-WS	9.63c	9.25a	7.20c	4.84a	0.34c	0.15a	322.30a	248.00b	30.33a	21.75a
Mean	13.22	8.25	8.87	4.92	0.44	0.15	310.96	267.81	28.10	18.10
**Yuxiangyouzhan**										
CK	17.01a	10.83a	12.72a	6.68b	0.71b	0.28b	316.81b	289.33b	29.55b	20.58b
WS	15.86a	10.07a	11.55a	5.17c	0.59c	0.18c	313.39b	262.67c	28.15c	21.65b
LL	13.98b	12.00a	11.15ab	8.49a	0.83a	0.41a	331.25a	317.00a	33.43a	26.88a
LL-WS	11.50c	11.61a	9.48b	5.70bc	0.57c	0.19c	329.12a	249.00c	33.60a	24.80a
Mean	14.58	11.13	11.22	6.51	0.67	0.27	322.64	279.50	31.20	23.50

Within a column for each cultivar, means followed by different letters are significantly different according to LSD (0.05).

CK, natural light and well-watered treatment; WS, natural light and water-stressed treatment; LL, low light and well-watered treatment; LL-WS, low light and water-stressed treatment; AS, After shading; MS, Maturity stage.

### Effect of the light-water treatments on antioxidant response and MDA content

Compared with CK, LL and LL-WS significantly increased SOD activity at AS, while the light-water treatments substantially decreased SOD activity at MS in Xiangyaxiangzhan. For Yuxiangyouzhan, WS and LL significantly increased SOD activity at AS. LL-WS significantly increased SOD activity, but LL significantly decreased SOD activity at MS. For Xiangyaxiangzhan, WS and LL-WS significantly reduced POD activity at AS and MS compared to that under CK. LL significantly decreased the POD activity at AS but significantly increased the POD activity at MS. Compared with CK, WS and LL-WS significantly increased the POD activity at AS, and the light-water treatments substantially increased the POD activity at MS in Yuxiangyouzhan. WS and LL-WS significantly increased the CAT activity at AS while LL and LL-WS significantly increased the CAT activity at MS in Xiangyaxiangzhan. For Yuxiangyouzhan, light-water treatments significantly increased the CAT activity at AS and MS. Compared with CK, LL and LL-WS significantly increased the MDA content at AS while WS and LL-WS significantly decreased the MDA content at MS in Xiangyaxiangzhan. For Yuxiangyouzhan, WS and LL significantly decreased the MDA content at AS. LL and LL-WS significantly increased the MDA content but WS significantly decreased the MDA content at MS compared to that under CK (Table 4).

**Table 4:** Effect of light-water treatment on antioxidant response and MDA content in leaves.

Treatment	SOD activity (U g^−1^ FW)		POD activity (U g^−1^ FW)		CAT activity (U g^−1^ FW)		MDA Content (μmol g^−1^FW)	
	AS	MS	AS	MS	AS	MS	AS	MS
**Xiangyaxiangzhan**								
CK	141.60c	256.94a	86.26a	131.21b	23.58b	41.36b	7.35c	7.16a
WS	139.14c	176.14b	66.95bc	113.36d	31.89a	50.22b	7.94bc	5.33c
LL	172.32b	167.27b	60.65c	151.85a	20.77b	79.02a	8.68b	6.76ab
LL-WS	214.04a	138.94c	74.99b	123.97c	32.81a	84.82a	9.88a	6.02bc
Mean	166.78	184.82	72.21	130.10	27.26	63.86	8.46	6.32
**Yuxiangyouzhan**								
CK	123.76b	194.19b	61.26b	70.58c	37.14b	31.67c	14.39a	4.88c
WS	186.47a	206.48b	70.77a	90.33b	54.61a	41.28b	11.06b	4.37d
LL	179.54a	172.72c	62.32b	87.39b	49.82a	43.27b	9.78b	6.75a
LL-WS	113.91b	239.50a	75.29a	132.30a	53.25a	76.09a	16.60a	5.25b
Mean	150.92	203.22	67.41	95.15	48.71	48.08	12.96	5.31

Within a column for each cultivar, means followed by different letters are significantly different according to LSD (0.05).

CK, natural light and well-watered treatment; WS, natural light and water-stressed treatment; LL, low light and well-watered treatment; LL-WS, low light and water-stressed treatment; AS, After shading; MS, Maturity stage.

### Effect of the light-water treatments on the 2AP content

Higher 2AP content in the grains was observed under LL and LL-WS than under CK. LL and LL-WS significantly increased the 2AP content in Yuxiangyouzhan by 18.67% and 16.32%, respectively, compared with that under CK. The WS significantly decreased the 2AP content in grains of Xiangyaxiangzhan and Yuxiangyouzhan by 24.44% and 7.19%, respectively, compared with that under CK (Table 5).

**Table 5:** Effect of light-water treatment on 2AP content in grains.

Treatment	2AP Content (μg g^−1^)	
	Xiangyaxiangzhan	Yuxiangyouzhan
CK	7.08a	7.23b
WS	5.35b	6.71c
LL	7.50a	8.58a
LL-WS	7.44a	8.41a
Mean	6.84	7.73

Within a column for each cultivar, means followed by different letters are significantly different according to LSD (0.05).

CK, natural light and well-watered treatment; WS, natural light and water-stressed treatment; LL, low light and well-watered treatment; LL-WS, low light and water-stressed treatment.

### Effects of the light-water treatments on P5C content, proline content, and GABA content

Compared with CK, WS significantly increased the P5C content in leaves at AS for Xiangyaxiangzhan and at MS for Yuxiangyouzhan. For Xiangyaxiangzhan, LL significantly decreased the P5C content in leaves at AS, and LL and LL-WS significantly increased the P5C content in leaves at MS. WS significantly decreased the P5C content in grains at AS for Xiangyaxiangzhan, but significantly increased the P5C content in grains at AS for Yuxiangyouzhan compared with those in the control. For Xiangyaxiangzhan, WS and LL-WS significantly increased the P5C content in grains at AS but significantly reduced the P5C content in grains at MS (Table 6).

**Table 6:** Effect of light-water treatment on P5C content, proline content and GABA content, soluble protein content in leaves and grains.

Treatment	P5C content (μmol g^−1^)				Proline content (μg g^−1^)				GABA content (mg g^−1^FW)				Soluble protein content (μg g^−1^ FW)			
	Leaves		Grains		Leaves		Grains		Leaves		Grains		Leaves		Grains	
	AS	MS	AS	MS	AS	MS	AS	MS	AS	MS	AS	MS	AS	MS	AS	MS
**Xiangyaxiangzhan**																
CK	1.19b	1.90c	1.60b	0.37a	48.06d	60.68b	20.14ab	7.07b	2.12a	1.48b	0.92b	0.71b	7.51b	7.86b	7.36a	7.14a
WS	1.35a	2.03bc	1.15c	0.42a	70.07b	72.20a	18.75ab	11.84a	1.77b	1.59b	1.07a	0.84ab	7.56ab	7.87b	7.32a	7.13a
LL	1.05c	2.15b	2.12a	0.21c	58.14c	62.57b	17.64b	8.69b	1.58b	2.14a	1.12a	0.87a	7.61ab	8.08a	7.34a	7.20a
LL-WS	1.27ab	2.47a	2.28a	0.29b	106.08a	72.61a	21.06a	11.43a	2.09a	1.32b	1.16a	0.80ab	7.61a	7.90b	7.35a	7.19a
Mean	1.22	2.14	1.79	0.32	70.58	67.02	19.40	9.76	1.89	1.63	1.07	0.81	7.57	7.93	7.34	7.17
**Yuxiangyouzhan**																
CK	1.55a	2.39b	1.23b	0.19a	34.79b	33.10b	16.88c	10.14a	1.27b	1.33ab	0.64b	0.54b	7.64a	7.75b	7.29a	7.05a
WS	1.52a	2.80a	1.59a	0.21a	40.16b	45.36a	27.80b	12.01a	1.48b	1.22bc	1.01a	0.67a	7.64a	7.81b	7.28a	7.14a
LL	1.57a	2.48b	1.13b	0.19a	38.94b	34.61b	32.47a	9.98a	1.49b	0.98c	1.18a	0.59b	7.62a	7.79b	7.32a	7.04a
LL-WS	1.62a	2.50b	1.41ab	0.20a	54.51a	45.08a	29.36ab	10.08a	2.11a	1.50a	1.03a	0.71a	7.64a	8.02a	7.44a	7.02a
Mean	1.57	2.54	1.34	0.19	42.1	39.54	26.63	10.55	1.59	1.26	0.96	0.63	7.64	7.84	7.33	7.06

Within a column for each cultivar, means followed by different letters are significantly different according to LSD (0.05).

CK, natural light and well-watered treatment; WS, natural light and water-stressed treatment; LL, low light and well-watered treatment; LL-WS, low light and water-stressed treatment; AS, After shading; MS, Maturity stage.

The light-water treatments significantly increased the proline content in leaves at AS in Xiangyaxiangzhan compared with that in CK. WS and LL-WS significantly increased the proline content in leaves at MS in both varieties. LL-WS significantly increased the proline content in leaves at AS in Yuxiangyouzhan compared with that under CK. The light-water treatments significantly increased the proline content in grains at AS in Yuxiangyouzhan. The WS and LL-WS significantly increased the proline content in grains at MS in Xiangyaxiangzhan compared with that under CK (Table 6).

Compared with CK, WS and LL significantly reduced the GABA content in leaves, while LL significantly increased the GABA content in leaves at MS in Xiangyaxiangzhan. For Yuxiangyouzhan, LL-WS significantly increased the GABA content in leaves at AS but LL significantly decreased the GABA content in leaves at MS. The GABA content in Xiangyaxiangzhan and Yuxiangyouzhan grains at AS was noticeably increased by the light-water treatments. LL significantly increased the GABA content in grains at MS in Xiangyaxiangzhan compared with that under CK. WS and LL-WS significantly increased the GABA content in grains at MS in Yuxiangyouzhan compared with that under CK (Table 6).

Compared with CK, LL-WS and LL significantly increased the soluble protein content in leaves at AS and MS in Xiangyaxiangzhan, respectively. LL-WS significantly increased the soluble protein content in leaves at MS in Yuxiangyouzhan. The soluble protein content in grains was not noticeably affected by the light-water treatments at AS or MS. (Table 6).

### Effect of the light-water treatments on P5CS, PDH, OAT, and DAO activity

Compared with CK, WS significantly increased the P5CS activity in leaves at AS and MS in Xiangyaxiangzhan and Yuxiangyouzhan. LL-WS resulted in a significant increase in P5CS activity in leaves at AS in Xiangyaxiangzhan and at MS in Yuxiangyouzhan. WS and LL-WS significantly increased the P5CS activity in grains at AS in Yuxiangyouzhan and at MS in Xiangyaxiangzhan. Compared with CK, LL and LL-WS significantly increased the P5CS activity in grains at MS in Yuxiangyouzhan (Table 7).

**Table 7:** Effect of light-water treatment on P5CS activity, PDH activity, OAT activity and DAO activity in leaves and grains.

Treatment	P5CS activity (U g^−1^ FW)				PDH activity (U g^−1^ FW)				OAT activity (U g^−1^ FW)				DAO activity (U g^−1^ FW)			
	Leaves		Grains		Leaves		Grains		Leaves		Grains		Leaves		Grain	
	AS	MS	AS	MS	AS	MS	AS	MS	AS	MS	AS	MS	AS	MS	AS	MS
**Xiangyaxiangzhan**																
CK	30.15c	51.31bc	4.03ab	2.71b	23.18a	24.96ab	33.92b	32.26b	20.98a	13.98c	38.92ab	34.51b	6.04a	7.61b	6.13a	5.56a
WS	44.01a	58.41a	3.69b	3.44a	18.4b	27.16a	47.85a	36.19a	10.95c	16.95b	41.52a	35.86ab	6.72a	8.20ab	6.48a	6.14a
LL	30.15c	55.52ab	4.60a	2.33b	17.56b	18.27c	33.02b	33.68ab	9.41c	19.27a	39.54a	35.63ab	5.41a	7.23b	6.26a	6.17a
LL-WS	35.28b	48.24c	3.73b	3.41a	15.85b	21.76bc	35.75b	34.74ab	14.46b	17.06b	35.75b	37.66a	6.29a	9.64a	6.26a	5.68a
Mean	34.90	53.37	4.01	2.97	18.75	23.04	37.63	34.22	13.95	16.81	38.94	35.92	6.11	8.17	6.28	5.89
**Yuxiangyouzhan**																
CK	39.27b	45.25c	2.32c	2.00b	20.27a	28.24a	28.61a	31.68b	21.93b	17.28a	38.60b	33.95a	6.89b	10.09ab	6.26b	5.52ab
WS	49.14a	53.02a	2.86b	1.75b	20.22a	27.57a	31.89a	32.90ab	17.57c	18.77a	44.85a	33.23a	8.54a	9.47bc	6.58a	5.48ab
LL	37.77b	45.03c	2.38bc	2.85a	20.43a	26.78a	31.39a	33.76a	26.50a	20.16a	39.60b	27.12b	8.31ab	8.23c	5.94c	5.39b
LL-WS	40.60b	49.31b	3.61a	2.84a	18.17a	32.33a	29.43a	33.49a	11.44d	20.14a	37.20b	31.92a	8.92a	11.14a	6.48ab	5.92a
Mean	41.70	48.15	2.79	2.36	19.77	28.73	30.33	32.96	19.36	19.09	40.06	31.55	8.16	9.73	6.31	5.58

Within a column for each cultivar, means followed by different letters are significantly different according to LSD (0.05).

CK, natural light and well-watered treatment; WS, natural light and water-stressed treatment; LL, low light and well-watered treatment; LL-WS, low light and water-stressed treatment; AS, After shading; MS, Maturity stage.

The PDH activity in leaves at AS in Xiangyaxiangzhan was significantly decreased under the light-water treatments compared to that under CK. LL significantly reduced the PDH activity in leaves at MS in Xiangyaxiangzhan. The PDH activity in leaves at AS and MS in Yuxiangyouzhan was not significantly affected by the light-water treatments. The WS significantly increased the PDH activity in grains at AS and MS in Xiangyaxiangzhan, while LL and LL-WS significantly increased the PDH activity in grains at MS in Yuxiangyouzhan compared with that under CK (Table 7).

Compared with CK, the light-water treatments significantly decreased the OAT activity in leaves at AS but significantly increased the OAT activity in leaves at MS in Xiangyaxiangzhan. For Yuxiangyouzhan, WS and LL-WS significantly reduced the OAT activity in leaves at AS but LL significantly increased the OAT activity in leaves at AS. The OAT activity in grains at MS was significantly increased under LL-WS in Xiangyaxiangzhan. The WS resulted in a significant increase in the OAT activity in grains at AS, but LL significantly decreased the OAT activity in grains at MS in Yuxiangyouzhan compared with that under CK (Table 7).

Compared with CK, LL-WS significantly increased the DAO activity in leaves at MS in Xiangyaxiangzhan. For Yuxiangyouzhan, WS and LL-WS significantly increased the DAO activity in leaves at AS, while LL significantly decreased the DAO activity in leaves at MS. The WS significantly increased the DAO activity in grains at AS, while LL significantly decreased the DAO activity in grains at AS in Yuxiangyouzhan compared with that under CK. The DAO activity in grains at AS and MS in Xiangyaxiangzhan was not significantly affected by the light-water treatments (Table 7).

### Effects of the light-water treatments on Na, Mg, Mn, and Fe contents

Compared with CK, WS significantly reduced the Na content in leaves at AS while LL and LL-WS significantly increased the Na content in leaves at AS in Xiangyaxiangzhan. The Na content in Xiangyaxiangzhan leaves at MS was significantly increased under the light-water treatments. For Yuxiangyouzhan, WS and LL-WS significantly decreased the Na content in leaves at AS, while LL and LL-WS significantly increased the Na content in leaves at AS and MS, respectively. The Na content in grains at AS and MS was significantly reduced under WS and LL-WS in Xiangyaxiangzhan while LL-WS significantly reduced the Na content in grains at MS in Yuxiangyouzhan compared with that under CK (Table 8).

**Table 8:** Effect of shading and water stress on Na, Mg, Mn, Fe content in leaves and grains.

Treatment	Na content (mg kg^−1^)				Mg content (ug kg^−1^)				Mn content (mg kg^−1^)				Fe content (mg kg^−1^)			
	Leaves		Grains		Leaves		Grains		Leaves		Grains		Leaves		Grain	
	AS	MS	AS	MS	AS	MS	AS	MS	AS	MS	AS	MS	AS	MS	AS	MS
**Xiangyaxiangzhan**																
CK	486.02b	409.43c	388.50a	197.99a	199.79a	169.04a	160.72b	146.32a	591.68c	576.59c	56.10c	83.58c	215.90a	152.53a	168.20a	15.06c
WS	469.72c	427.80b	369.51b	179.47b	205.08a	173.13a	169.49a	148.27a	695.85a	924.72a	90.79a	132.98a	213.78a	114.07b	160.83b	69.03a
LL	521.49a	456.01a	374.80ab	198.00a	207.86a	170.57a	158.28b	147.03a	559.98d	563.71c	49.66d	90.51c	194.71b	159.24a	161.29b	43.85b
LL-WS	534.46a	431.34b	342.67c	175.62b	205.54a	170.78a	163.71ab	144.84a	649.45b	739.79b	68.04b	114.19b	178.75b	117.96b	158.87b	24.39c
Mean	502.92	431.15	368.87	187.77	204.57	170.88	163.05	146.61	624.24	701.20	66.15	105.31	200.79	135.95	162.30	38.08
**Yuxiangyouzhan**																
CK	524.10b	430.22b	400.40a	188.90a	210.49a	172.98bc	166.99ab	147.07a	526.64c	534.70d	57.67a	48.10c	180.58bc	153.36b	201.69a	18.10a
WS	478.14c	450.23b	395.09a	178.70ab	212.97a	186.10a	160.84b	148.32a	594.88a	688.05a	49.04b	89.10a	194.65b	166.12a	175.54b	12.72b
LL	548.12a	444.25b	411.01a	174.90ab	209.60a	168.16c	168.83a	148.54a	566.73b	583.50b	19.50d	42.67d	435.78a	135.09c	195.11a	11.62c
LL-WS	477.21c	474.82a	401.94a	161.10b	205.94a	180.28ab	163ab	147.94a	587.08a	571.00c	32.05c	65.54b	158.58c	127.31c	170.52b	17.80a
Mean	506.90	449.88	402.11	175.90	209.75	176.88	164.92	147.97	568.83	594.31	39.56	61.35	242.40	145.47	185.71	15.06

Within a column for each cultivar, means followed by different letters are significantly different according to LSD (0.05).

CK, natural light and well-watered treatment; WS, natural light and water-stressed treatment; LL, low light and well-watered treatment; LL-WS, low light and water-stressed treatment; AS, After shading; MS, Maturity stage.

The Mg content in Xiangyaxiangzhan leaves at AS and MS was not significantly affected by the light-water treatments. For Yuxiangyouzhan, WS significantly increased the Mg content in leaves at MS compared to that under CK. WS significantly increased the Mg content in Xiangyaxiangzhan grains at AS. The light-water treatments did not significantly affect the Mg content in Yuxiangyouzhan grains (Table 8).

Compared with CK, WS and LL-WS significantly increased the Mn content in leaves and grains at AS and MS but LL significantly decreased the Mn content in leaves and grains at AS in Xiangyaxiangzhan. For Yuxiangyouzhan, the light-water treatments significantly increased the Mn content in leaves at AS and MS, and the Mn content in grains at AS was significantly decreased under the light-water treatments compared with that in the control. WS and LL-WS significantly increased the Mn content in grains at MS, but LL significantly decreased the Mn content in grains at AS (Table 8).

Compared with CK, for Xiangyaxiangzhan, LL and LL-WS significantly decreased the Fe content in leaves at AS, while WS and LL-WS significantly decreased the Fe content in leaves at MS. For Yuxiangyouzhan, LL and WS significantly increased the Fe content in leaves at AS and MS, respectively. LL and LL-WS significantly decreased the Fe content in Yuxiangyouzhan leaves at MS. For Xiangyaxiangzhan, the light-water treatments significantly decreased the Fe content in grains at AS. WS and LL significantly increased the Fe content in grains at MS in Xiangyaxiangzhan but significantly decreased the Fe content in grains at MS in Yuxiangyouzhan. WS and LL-WS significantly decreased the Fe content in grains at AS in Yuxiangyouzhan compared with that under CK (Table 8).

### Correlation analysis

**Figure 1:**
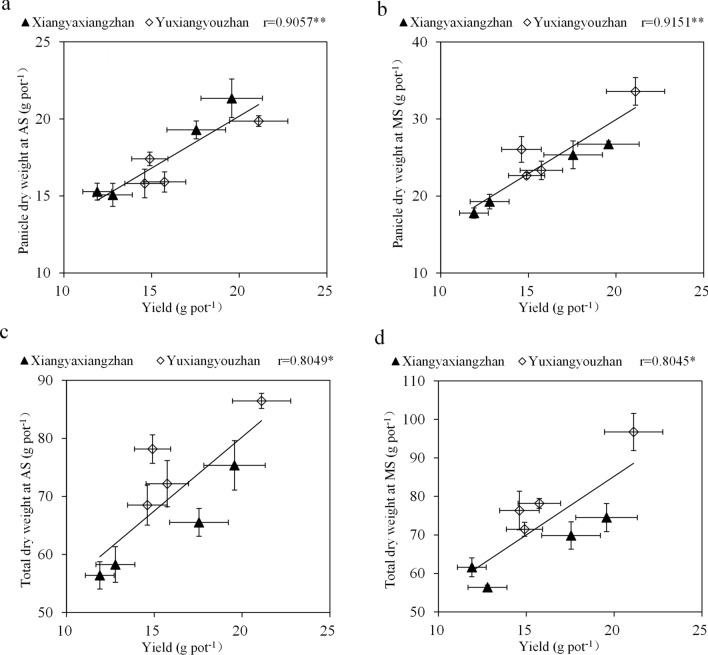
Correlation analyses between grain yield and plant dry weight. * and **, significant at the 0.05 and 0.01 probability levels, respectively.

**Figure 2:**
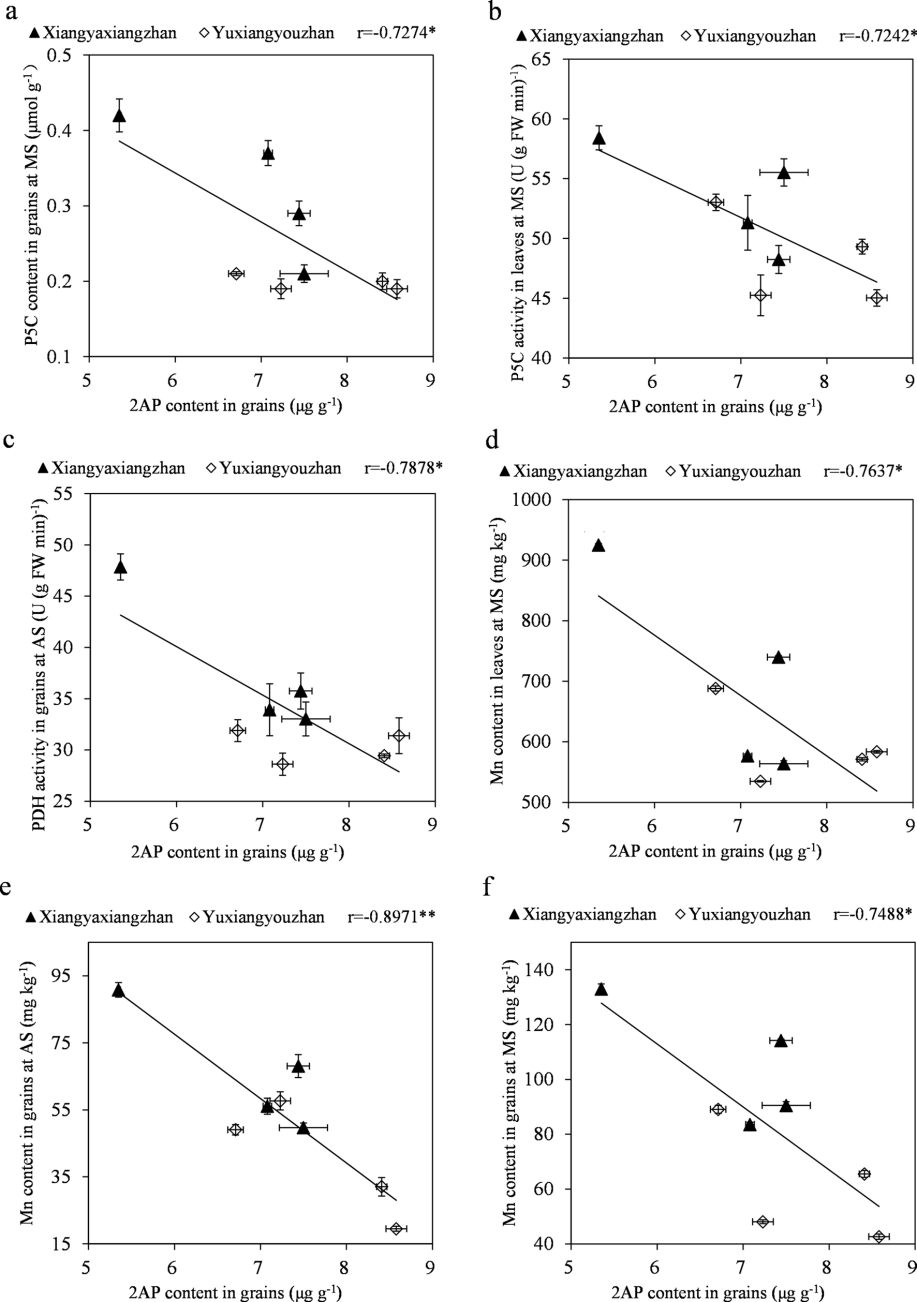
Correlation analysis 2AP content and physiological parameters and element content. * and **, significant at the 0.05 and 0.01 probability levels, respectively.

There was a significant positive correlation between the grain yield and the panicle dry weight and the total dry weight at AS and MS (Fig. 1). The 2AP content in grains was significantly negatively correlated with the P5C content in grains, P5CS activity in leaves at MS, and PDH activity in gains at AS. The Mn content in leaves at MS and in grains at AS and MS showed a significant positive correlation with the 2AP content in grains (Fig. 2).

## Discussion

The effects of low light and water stress on grain yield in rice have been reported^19,21,34,35^. Shading and water stress have a negative significant effect on the total dry weight of rice^19,26^. In this study, we confirmed that low light reduced the yield of rice mainly by reducing the filled grain percentage and the total dry weight (Tables 1 and 2). This study found a significant positive correlation between grain yield and the dry weight of the panicle and total dry weight (Fig. 1). The light-water treatments had no significant effect on the panicle number or 1,000-grain weight (Table 1), this finding is consistent with a previous report in which the 1,000-grain weight was not affected by a shading treatment during early grain filling^35^ but is different from the result of another study due to the difference in the shading duration^19^. Shading resulted in a reduction in the number of effective panicles, and the extent of the reduction varies depending on the treatment period^19,21,36^. A significant reduction in effective panicles could be observed at the tillering stage^36^. Many studies have shown that water stress resulted in a significant reduction in the filled grain percentage towards the mid-tillering, booting and flowering stages^37,38^. In this study, the light-water stress treatment reduced the grain yield and the filled grain percentage (Table 1).

Studies have reported that shading significantly increased the total chlorophyll content of plants^39,40^. In this study, a significant increase in the SPAD value in response to light-water treatments was observed in Xiangyaxiangzhan. For Yuxiangyouzhan, WS significantly decreased the SPAD value, while LL and LL-WS significantly increased the SPAD value at MS (Table 3). Leaf gas exchange is important for plants in response to abiotic stress^41^. Studies have reported that low light and water deficits caused a change in Pn, Tr, Gs, and Ci^22,42–47^. In this study, the light-water treatments affected the gas exchange parameters after the shading treatment, and at the maturity stage, the effect varied between varieties (Table 3). The differences in the changes in SPAD and gas exchange parameters were mainly due to the time and degree of the shading and water stress treatments.

Shading and water stress both result in the accumulation of reactive oxygen species (ROS) and cause damage to proteins and lipids^48–50^. Superoxide dismutase (SOD), peroxidase (POD) and catalase (CAT) are key enzymes used for scavenging reactive oxygen species; MDA is the product of lipid peroxidation in cells and reflects the extent of cell membrane damage under stressful conditions^48,51,52^. Shading significantly reduced SOD activity and increased MDA content during the grain filling stage^22^. Shade tolerant varieties maintain a lower MDA content and higher SOD, POD, and CAT activity and soluble protein content^50^. Moreover, the MDA content was significantly increased and the activities of SOD and CAT were significantly reduced after a PEG treatment^49^, which may have been due to the drought-induced accumulation of H_2_O_2_ in the guard cells^53^. In this study, the low light treatments significantly increased the SOD activity and MDA content in both rice varieties. However, different changes in CAT activity and POD activity after shading were observed in the two varieties. At the maturity stage, the shading treatment resulted in a significant reduction in SOD activity and increased POD and CAT activity in Yuxiangyouzhan rice, while the MDA content was significantly increased (Table 4). The light-water treatments had a regulatory effect on the antioxidant response parameters. Further studies are needed to evaluate the molecular basis of the complex responses of rice plants to abiotic stress, i.e., light-water treatments^54^.

Many previous studies have reported that abiotic stresses increase the content of 2AP in grains^19,25,26^. Lower levels of water irrigation affected 2AP accumulation in aromatic rice^25,26^. The 2AP content in grains increased significantly after shading during the grain filling period^19^. In this study, low light treatments increased the 2AP content in grains of both varieties, but WS significantly decreased the content of 2AP in grains (Table 5). The responses of different genotypes to the levels of water stress may explain the difference in the changes in 2AP accumulation in this study and in previous studies^25,26^.

Shading significantly increased the GABA content in Yuxiangyouzhan and Nongxiang18 and increased the proline content in Yuxiangyouzhan grains^19^. The proline content in tomato was reduced in drought tolerant varieties^55^. Different water regimes coupled with nitrogen affect the biosynthesis of 2AP by regulating physiological and biochemical parameters such as the P5C, proline, and GABA content and the activity of P5CS, PDH, OAT, and DAO^26^. In this study, the light-water treatments regulated the P5C, proline, and GABA content in leaves and grains as well as the P5CS, PDH, OAT, and DAO activity in leaves and grains (Tables 6 and 7). The relationship of the 2AP content in grain to the studied physiological parameters was assessed (Fig. 2a–c). The relationship between the 2AP content and the 2AP-related physiological and biochemical parameters differed among experimental treatments and genotypes^56–58^. Moreover, the light-water treatments regulated the dynamics of the element content in leaves and grains (Table 8), and the relationship between the 2AP in grains and the element content was also assessed (Fig. 2d–f). Inconsistent results were obtained for the relationship between the 2AP content and the element content of different elements among different experimental treatments^59^. Moreover, element levels in plants and the deficits or excess elements such as iron in plants are related to oxidative stress in plants^60,61^. Therefore, element absorption regulated by the light-water treatments further influenced oxidative stress in the rice plants, which resulted in more complex changes in the metabolic physiology of the plants. Further studies on the molecular basis of 2AP biosynthesis regulation in aromatic rice under light-water treatments should be conducted.

Overall, light-water treatments during the early grain filling stage regulate yield and 2AP formation, which results from biomass accumulation, photosynthesis, antioxidant responses, 2AP formation related physiological attributes, and element absorption in the plant.

## Conclusion

Light-water treatments during the early grain filling stage regulates yield by affecting the plant dry weight, gas exchange parameters, and antioxidant responses. However, these treatments also influence 2AP accumulation by regulating 2AP formation-related physiological parameters and elemental levels. Further study is needed to balance yield with 2AP accumulation under light-water treatments.

## Methods

### Experimentation and treatments

A pot experiment was conducted at the Experimental Farm, South China Agricultural University, Guangzhou, China during July–November 2017. This region is favourable for the growth of aromatic rice due to its humid subtropical monsoon climate. Two aromatic rice varieties, Yuxiangyouzhan and Xiangyaxiangzhan, were used in this study. The two varieties are popular aromatic rice cultivars in South China. The soil used for the experiment was collected from paddy fields^19^.

**Figure 3:**
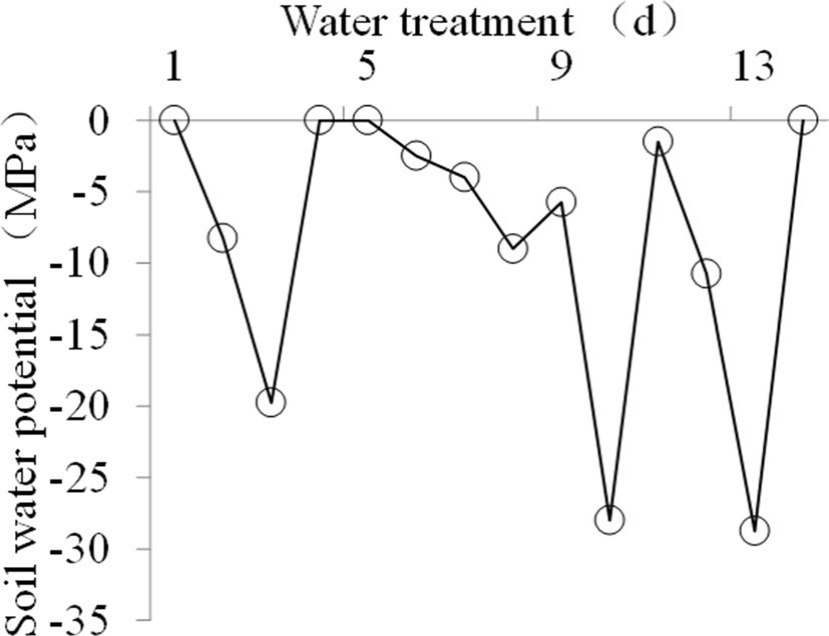
Soil water potential for the WS treatment.

Two light levels (natural light and low light) were employed in this study. The low light treatment was implemented with a black netting layer and was equivalent to a 67% reduction in the full natural light level^19^. Two water treatments, well-watered and water-stressed, were conducted in this study (Fig. 3). The water stress treatment was conducted according to the method described in a previous study^62^. The well-watered treatment was flooded to a depth of 1–2 cm by manually adding tap water^63^. Four light-water treatments (CK: natural light and well-watered treatment, WS: natural light and water-stressed treatment, LL: low light and well-watered treatment, LL-WS: low light and water-stressed treatment) were conducted during the early grain filling stage. The treatments lasted for 15 days, from September 26th to October 10th.

Seeds of the two aromatic rice varieties were sown on July 15th and 15-day-old seedlings were transplanted into pots with four seedlings per hill and five hills per pot. A compound fertilizer (15:15:15) was applied basally in the amount of 5.5 g per pot. The rice plants were harvested on November 6th. Except during the water treatment period, the irrigation was carried out according to routine management practices: a 2–4 cm water layer was maintained from transplanting to 7 days before harvest, and then the soil was allowed to dry out naturally. Other managements practices were the same in all treatments and followed local recommendations.

### Sampling and measurement

#### Determination of yield and yield-related traits and dry matter weight

The determination of yield and yield-related traits was performed according to a previously reported method^64^. At the maturity stage (MS), four pots were randomly harvested from each treatment. The grains were sun-dried to a moisture content of 14%. The effective panicles per pot were determined by counting the panicle numbers in four pots from each treatment. The grain number per panicle and the filled grain number were counted in the same four pots, and the filled grain percentage was calculated. The 1,000-grain weight was measured by weighing 1,000 grains from four random samples. Six representative plants were selected randomly and taken to the laboratory. The plants were separated into their panicles, leaves, and stem sheaths and then dried at 80 °C to a constant weight.

#### Determination of gas exchange parameters and SPAD value

The net photosynthetic rate (Pn), transpiration rate (Tr), stomatal conductance (Cond) and intercellular CO_2_ concentration (Ci) of the leaf blades were determined with an LI-6400XT portable photosynthesis system (LI-COR, Inc., USA) after shading and at maturity from 9:00 am to 11:00 am on sunny days, and four measurements were taken for each treatment. Meanwhile, the SPAD value was measured by a SPAD meter ‘SPAD-502′ (Konica Minolta, Japan), with four replications for each treatment.

#### Determination of malondialdehyde (MDA) and antioxidant activities

The malondialdehyde (MDA) and antioxidant activities were measured as described method by Li et al.^65^. MDA was reacted with thiobarbituric acid (TBA), and the absorbance of the reaction solutions was recorded at 532 nm, 600 nm, and 450 nm. The MDA content was expressed as μmol g^−1^ FW. The superoxide (SOD, EC 1.15.1.1) activity was measured by using the nitro-blue tetrazolium (NBT) method. The reaction mixture contained 1.75 ml of sodium phosphate buffer (pH 7.8), 0.3 ml of 130 mM methionine buffer, 0.3 ml of 750 μmol NBT buffer, 0.3 ml of 100 μmol EDTA-Na_2_ buffer, 0.3 ml of 20 μmol lactoflavin and 0.05 ml of enzyme extract. After the reaction, the change in colour was measured at 560 nm. The SOD activity was expressed as U g^−1^ FW. For peroxidase (POD EC1.11.1.7) activity, the enzyme extract (50 μl) was added to the reaction solution containing 1 ml of 0.3% H_2_O_2_, 0.95 ml of 0.2% guaiacol, and 1 ml of 50 mM sodium phosphate buffer (pH 7.0). The absorbance was read at 470 nm. The POD activity was expressed as U g^−1^ FW. For the catalase (CAT, EC 1.11.1.6) activity, an aliquot of enzyme extract (50 μl) was added to the reaction solution containing 1 ml of 0.3% H_2_O_2_ and 1.95 ml of sodium phosphate buffer, and the absorbance was recorded at 240 nm. The CAT activity was expressed as U g^−1^ FW.

#### Determination of 2AP concentration

The 2AP concentration in the grains was measured using a previously described procedure^19,66^ that used the synchronization, distillation and extraction method (SDE) combined with GC–MS–QP 2010 Plus system (Shimadzu Corporation, Japan).

#### Determination of 2AP formation related to physiological traits

Fresh samples of grains and flag leaves were collected from each plot and immediately stored at − 80 °C until the determination of the 1-pyrroline-5-carboxylic acid (P5C) content, proline content, soluble protein content, γ-aminobutyric acid (GABA) content, proline dehydrogenase (PDH) activity, pyrroline-5-carboxylic acid syntheses (P5CS) activity, ornithine aminotransferase (OAT) activity, and diamine oxidase (DAO) activity.

The P5C concentration was determined according to a previously described method^67^. The reaction mixture consisted of 0.2 ml of enzyme extraction supernatant, 0.5 ml of 10% trichloroacetic acid (TCA), and 0.2 ml of 40 mM 2-aminobenzaldehyde. The absorbance was measured at 440 nm after the reaction, and the P5C concentration was expressed as μmol g^−1^ FW. The proline content was evaluated by using a previously reported method^68^. The proline content was expressed as μg g^−1^ FW. The soluble protein content was determined according to a previously reported method^69^ with G-250. The soluble protein content was expressed as μg g^−1^ FW. The GABA content was measured according to previously described methods^70,71^. Plant tissue (0.500 g) was homogenized with 60% ethanol (5 ml) and then oscillated for 4 h in an oscillations instrument (HZS-H, China) at 200 oscillations per minute. Then, the supernatant was centrifuged at 8,000 rpm for 3 min. The reaction mixture in a 10 ml test tube consisted of 1 ml of the supernatant, 0.6 ml of 0.2 M (pH 9.0) sodium tetraborate, 2 ml of 5% toluene and 1 ml of 7% sodium hypochlorite. The prepared mixture was heated in a boiling water bath for 5 min and then cooled. The absorbance of the reaction mixture was measured at 645 nm. The GABA content was expressed as μg g^−1^ FW.

The activity of PDH was measured by following a previously described method^72^. After the reaction, the absorbance was read at 440 nm, and the PDH activity was expressed as U g^−1^ FW. The P5CS activity was determined according to a reported method^73^. The reaction solutions contained 10 mM ATP, 20.0 mM MgCl_2_, 50 mM Tris–HCl buffer, 50 mM sodium glutamate, 100 mM hydroxamate-HCL and 0.5 ml of enzyme extract. The prepared mixture was kept in a 37℃ water bath for 5 min, and then the reaction was terminated by the addition of 0.5 ml of a stop buffer (2.5% FeCl_3_ and 6% TCA, dissolved in 100 ml of 2.5 M HCl). The P5CS activity was expressed as U g^−1^ FW. The activity of OAT was assayed by using a previously described method^71^. The absorbance of the supernatant fraction was read at 440 nm. The OAT activity was expressed as U g^−1^ FW. The DAO activity was measured according to previously reported methods^74,75^. The DAO activity was expressed as U g^−1^ FW.

#### Determination of the Na, Mg, Mn, and Fe contents in leaves and grains

Briefly, the plant tissue (leaves and grains) was oven-dried and ground into a fine powder. Then 0.30 g of the plant tissue sample was digested with a 10 ml diacidic mixture of HNO_3_:HClO_4_ (4:1 v/v), after which the resultant solutions were diluted to 25 ml. The Na, Mg, Mn, and Fe contents in leaves and grains were estimated by using an atomic absorption spectrophotometer (AA6300C, Shimadzu, Japan)^59^.

### Statistics

Analysis of variance (ANOVA) and correlation coefficients were performed using Statistix version 8 (Analytical, Tallahassee, Florida, USA). The differences amongst means separated by using the least significant difference (LSD) test at 5% significance level.

## Data Availability

All data generated or analyzed during this study are included in the article.
